# Small Vessel Disease/White Matter Disease of the Brain and Its Association With Osteoporosis

**DOI:** 10.14740/jocmr2119w

**Published:** 2015-03-01

**Authors:** Kannayiram Alagiakrishnan, Jenny Hsueh, Edwin Zhang, Khurshid Khan, Ambikaipakan Senthilselvan

**Affiliations:** a Department of Medicine, University of Alberta, Edmonton, Alberta, Canada; b Department of Radiology and Diagnostic Imaging, University of Alberta, Edmonton, Alberta, Canada; c School of Public Health, University of Alberta, Edmonton, Alberta, Canada

**Keywords:** White matter disease severity, Small vessel cerebral disease, Bone remodeling, Osteoporosis, Vascular risk factors

## Abstract

**Background:**

Evidence now suggests the role of neural effect on bone mass control. The effect of small vessel disease of the brain on osteoporosis has not been studied. The aim of this study was to investigate the association of white matter disease (WMD) of the brain with osteoporosis in the elderly.

**Methods:**

In this retrospective cross-sectional study, 780 consecutive patient charts between 2010 and 2011 were reviewed in the Senior’s Outpatient Clinic at the University of Alberta Hospital. Subjects with brain computerized tomography (CT) were included in the study. Subjects with incomplete information, intracranial hemorrhage, acute stroke, cerebral edema, and/or normal pressure hydrocephalus on the CT were excluded. WMD was quantified on CT using Wahlund’s scoring protocol. Osteoporosis information was obtained from the chart, which has been diagnosed based on bone mineral density (BMD) information. Logistic regression analysis was done to determine the association of WMD severity with osteoporosis after controlling for confounding vascular risk factors.

**Results:**

Of the 505 subjects who were included in the study, 188 (37%) had osteoporosis and 171 (91%) of these osteoporotic subjects were females. The mean age was 79.8 ± 7.04 years. The prevalence of WMD in osteoporosis subjects was 73%. In the unadjusted logistic regression analysis, there was a significant association between WMD severity and osteoporosis (odds ratio (OR): 1.10; 95% confidence interval (CI): 1.05 - 1.14; P < 0.001) and the significance remained in the adjusted model, after correcting for age, sex and all vascular risk factors (OR: 1.11; 95% CI: 1.05 - 1.18; P < 0.001).

**Conclusion:**

WMD severity of the brain was associated with osteoporosis in the elderly.

## Introduction

Osteoporosis is a major public health concern and a common skeletal disease in the elderly. It is characterized by low bone mass with a consequent increase in fracture susceptibility. In the United States, approximately 10 million individuals have osteoporosis and approximately 34 million are at increased risk owing to low bone mass [1]. It is more commonly seen in women than in men. With aging the incidence of osteoporosis and resulting osteoporotic fractures also increases. Low bone mass and deterioration of the bone microarchitecture lead to fragility fractures. One study shows that the predicted lifetime risk of acquiring any fracture of the hip, spine, or distal forearm is 40% in Caucasian women over the age of 50 years [2]. Bone circulation and vascular risk factors play an important role in the pathophysiology of osteoporosis [3]. In postmenopausal women, atherosclerosis and impaired endothelial function have been associated with osteoporosis [4, 5]. Bone circulation is important for skeletal modeling and remodeling as well as in the healing process [6]. At present, growing evidence indicates the existence of a correlation between cardiovascular disease (atherosclerosis) and osteoporosis. These conditions have shared risk factors and pathophysiology [7, 8]. Observational and cross-sectional studies showed an association between low 25-hydroxy vitamin D levels and cardiovascular health [9, 10].

Evidence now also suggests the role of the nervous system on bone mass control [11, 12]. Studies have also shown that there is neurogenic control of bone metabolism through the interplay of central, peripheral and autonomic nervous system. Nerves in the bone are found in metabolically active areas and the majority of nerves are localized along blood vessels. The “neurovascular unit” is a term often used to describe brain blood vessels which are in intimate physical and functional connection between the brain (nervous) tissue and blood vessels. Neurotransmitters like neuropeptides and catecholamine are thought to play a key role in bone formation [13]. The interaction between the bones, brain, and the blood vessels may play a role in bone health and disease. So nervous system disorders can influence bone health [14]. One such nervous system disorder is cerebral white matter disease (WMD). This can occur as a result of small vessel disease of the brain. It is characterized by pathological changes seen in small arteries, arterioles, venules, and capillaries of the brain. These changes lead to ischemia mainly in the subcortical structures and can cause cerebral white matter changes (leukoaraiosis). WMD of the brain is a neuroimaging finding of cerebral small vessel disease. It was initially considered simply a normal age-related process, but recent studies indicate it is not an innocuous finding. These changes in the white matter are detected by computerized tomography (CT) and magnetic resonance imaging (MRI) of the brain [15]. WMDs are associated with vascular risk factors [16]. Ischemia plays an important role in the pathogenesis of WMD, leading to demyelination and astrocytic gliosis [17, 18].

Osteoporosis and cerebrovascular disease (CVD) are chronic diseases which have been considered to be independent and whose common characteristic is increasing incidence with age. Osteoporosis has been recognized as a complication after CVD. Several long-term prospective studies showed non-uniform pattern of changes in BMD, with significant loss on the paralysis side, especially with large vessel CVD [19, 20]. Central neurological disorders may also increase the risk of fracture by multiple pathways including changes in bone mineral density and quality of bone microarchitecture, as well as increased risk of falls [21]. A recent cross-sectional study in healthy adults showed asymptomatic cerebral small-vessel disease was associated with postural instability. This could be due to bone weakness leading to locomotion and balance problems. The common cause of bone weakness is osteoporosis [22].

Current evidence suggests that WMDs of the brain (small vessel CVD) are clinically important. A link between WMD of the brain and osteoporosis is possible as they share common vascular risk factors through atherosclerosis. No study has investigated the association of small vessel CVD/WMD with osteoporosis. So the objective of this study was to investigate the association between WMD of the brain and osteoporosis in the elderly.

## Methods

### Study design

The study was a retrospective chart review with a cross-sectional design. This study design is similar to a design published with our previous study [23]. Consecutive patient’s charts that attended the outpatient Senior’s Clinic at the University of Alberta Hospital between 2010 and 2011 were reviewed. CT brain was performed for various reasons: cognitive impairment/dementia, falls and mobility problems. Subjects who had CT scan of brain were included in the study. Subjects who had incomplete information or any CT findings that would mimic WMD such as hemorrhage, acute stroke, cerebral edema, and/or normal pressure hydrocephalus in CT of the brain were excluded from the study. Ethics approval was obtained from the University of Alberta Ethics Board.

### Osteoporosis

Osteoporosis information was obtained from the chart. The diagnosis was made from the bone mineral density (BMD) information.

### Assessment of WMD

CT-based rating scales have been used by several researchers for the same. One such valid and reliable scale has been devised by Wahlund et al which is a sensitive scale applicable for both CT and MRI [24]. There were two physicians, one neurologist and one radiologist, who performed the WMD volume rating. The radiologist rated all the cases, while the neurologist independently rated selected cases. Inter-rater reliability between the two physicians and intra-rater reliability of the radiologist were measured. The raters were blinded to the patients’ medical status and history. The intra-rater reliability of WMD volume scoring for all regions from the data collected by one rater (radiologist) on 30 randomly chosen CT brains on two separate occasions 14 days apart, determined by the Kappa statistic, was 0.90. The inter-rater reliability between the radiologist and neurologist for all regions was 0.86. Total possible WMD score based on Wahlund’s criteria is 30. In this study, WMD score of 0 was considered as absences of WMD, and WMD score of 1 and above was considered as having WMD.

### Clinical information

Demographic and clinical information were collected. The following data: age, sex, body mass index (BMI), history of smoking, alcoholism, hypertension (HTN), diabetes mellitus (DM), hyperlipidemia, coronary artery disease (CAD), heart failure (HF), atrial fibrillation (A.fib), peripheral vascular disease (PVD) and renal failure were also collected.

### Data analysis

The dependent variable was presence or absence of osteoporosis and was used as a dichotomous variable in the logistic regression. The independent variable was severity of WMD and this was used as a continuous variable. The goal of our analysis was to assess the independent association of WMD severity with osteoporosis.

The difference in the prevalence of osteoporosis was compared in subjects with and without WMD. The statistical significance of these differences was assessed using the Chi-square test for dichotomous variables and using *t*-test for continuous variables. Logistic regression analysis was done to determine the independent association of global WMD score with osteoporosis after controlling for age, sex and vascular risk factors. Adjusted odds ratio (OR) and their 95% confidence interval (CI) indicated the independent association of each variable after controlling for the confounding effects of the other variables in the model. All analyses were performed using SPSS and statistical significance was set at P < 0.05 for all statistical tests.

## Results

Of the 505 subjects included in the study, 326 (64.6%) were females. The mean age was 79.82 (SD 7.04), while 54% of subjects were more than 80 years old. One hundred eighty-eight subjects (37%) had osteoporosis and 171 (91%) of these osteoporotic subjects were females. In total, 401 (79.4%) subjects had WMD as indicated by a WMD score of 1 and above. Mean WMD for osteoporosis group was 6.19 (SD 4.74) and for the non-osteoporosis group was 4.37 (SD 4.09).

The characteristics of the subjects are shown in Table 1 for osteoporosis and non-osteoporosis groups. As shown in Table 1, the prevalence of WMD was significantly greater in osteoporosis (72.9%) than in the non-osteoporosis group (62.5%). There was significantly greater proportion of subjects with obesity, hyperlipidemia and coronary heart disease in the osteoporosis group than in the non-osteoporosis group (Table 1). Smoking was significantly more prevalent in the osteoporosis group than in the non-osteoporosis group (Table 1). In the univariate logistic regression analysis (Table 2), there was a significant association between WMD score with osteoporosis (OR: 1.10; 95% CI: 1.05 - 1.14; P < 0.001) and the significance remained (P < 0.001) after controlling for age and sex (Table 2) and after controlling for age, sex and all vascular risk factors (P < 0.001) (Table 2).

**Table 1:** Baseline Characteristics of the Study Variables

Factor	Osteoporosis present (n = 188)	Osteoporosis absent (n = 317)	P value
Age			
> 80 years	75 (39.9%)	180 (56.8%)	< 0.001
≤ 80 years	113 (60.1%)	137 (43.2%)	
Sex			
Females	171 (90.1%)	155 (48.8%)	< 0.001
Males	17 (9.1%)	162 (51.1%)	
BMI (kg/m^2^)			
≥ 26	83 (48.8%)	180 (61.0%)	< 0.01
Smoking			
Non-smoker	120 (64.5%)	139 (45.1%)	< 0.01
Past or current smoker	66 (35.5%)	169 (54.8%)	
Alcoholism	88 (47.6%)	164 (53.1%)	0.27
Diabetes Mellitus	35 (9.9%)	68 (21.5 %)	0.49
Hypertension	136 (72.3%)	220 (69.4%)	0.55
Hyperlipidemia	77 (40.9%)	168 (52.2%)	< 0.01
Coronary artery disease	28 (14.9%)	87 (27.4%)	< 0.01
Peripheral vascular disease	2 (1.1%)	11 (3.5%)	0.15
Atrial fibrillation	25 (13.3%)	43 (13.6%)	1.00
Heart failure	12 (6.4%)	19 (5.9%)	0.85
Renal failure	14 (7.4%)	23 (7.3%)	1.00
White matter disease prevalence	137 (72.9%)	198 (62.5%)	0.02
White matter disease severity	6.19 (SD 4.74)	4.37 (SD 4.09)	< 0.01

P values indicate the statistical significance between the presence and absence of WMD and the factors (per Chi-square analysis). BMI: body mass index.

**Table 2:** Logistic Regression Analysis: Global White Matter Disease Score and Its Association With Osteoporosis

Model	Odds ratio	95% confidence interval	P-value
Global white matter disease alone	1.10	1.05 - 1.14	< 0.001
Corrected for age and sex	1.10	1.04 - 1.15	< 0.001
Corrected for age, sex and vascular risk factors	1.11	1.05 - 1.18	< 0.001

White matter disease score was considered as a continuous variable.

## Discussion

This study shows an association between WMD (ischemic small vessel CVD) of the brain and osteoporosis in the elderly. There are numerous factors that may account for this relationship including risk factors that are common to both chronic conditions and varied pathophysiological mechanisms. The possible multiple mechanisms that can be responsible for the observed relationship between WMD and osteoporosis are discussed below.

WMD can affect neurons and neurotransmitters which have been shown to be involved in the bone homeostasis regulatory mechanisms [14]. In postmenopausal women, estrogen therapy has been shown to improve BMD [25]. Similarly, estrogen therapy has been shown to prevent reduction in cerebral blood flow after menopause as well as the development of CVD as evidenced by white matter lesions on MRI [26]. In the national risk assessment osteoporosis study, 80% of low-trauma osteoporotic fractures are seen in women who do not have osteoporosis [27]. The study of osteoporotic fractures found that 54% of postmenopausal women with hip fractures did not have osteoporosis on the hip [28]. Non-bone factors like decreased muscle strength and neurological disorders can play a role in fractures. This raises the possibility that the brain may have its influence through vascular biology on the bone. The effects may be mediated by the development of CVD, postural instability, muscle weakness and bone fragility. This study indirectly raises a possible link of cerebral WMD on bone.

The autonomic nervous system (ANS) plays a role in bone mass regulation and osteoporosis. ANS also regulates the blood vessels throughout the body. Bone is richly innervated by sensory and autonomic nerves. Continuous remodeling of the bone is a process consisting of bone resorption by osteoclasts and bone formation by osteoblasts [12]. Bone is a highly vascularized tissue and it receives 5-10% of cardiac output. Periosteal artery and nutrient artery system supply blood to the bones. Neural mechanisms control the bone circulation by providing nerve supply in the perivascular regions of the periosteum, metaphysis, epiphysis and cortical bone [29]. In a retrospective study on postmenopausal women, atherosclerotic vascular disease was most commonly seen with osteoporosis or osteopenia, compared to the control group with no osteoporosis or osteopenia (P < 0.001) [4]. In post-menopausal women, endothelial function impairment in the forearm has also been shown to be associated with low BMD. In this study, after adjustment for age, body mass index, years after menopause, and basal forearm blood flow, women with osteoporosis had a significantly lower maximal blood flow response to reactive hyperemia than with normal BMD (P = 0.029) [5]. Biological and epidemiological evidence support the link between (cardio) vascular disease and osteoporosis beyond age and shared risk factors [30-33]. Ischemic CVD may affect the ANS, which potentially increases the risk for further vascular disease in the body, including bone [34]. This is another mechanism by which WMD may affect bone remodeling and cause osteoporosis.

To our knowledge, this is the first study to show an association of small vessel disease (WMD) of the brain and osteoporosis. But this study has some limitations. One of the limitations of the study is its cross-sectional nature, which can give only the information about the association, but not the risk or causality. The study sample was a referred population to the Geriatric specialist clinic. Therefore the possibility of selection bias could not be excluded in this study and precludes generalization to a broad elderly population. We cannot exclude the potential role of vitamin D deficiency as a potential mediator of the observed association, since we do not have information on vitamin D status on all subjects. The Wahlund’s observational rating scale that was used provided a reliable cross-sectional assessment of WMD burden of the brain. But visual rating scales on non-contrast CT are less sensitive than measures of WMLs on MRI. Although CT is less sensitive to white matter changes than MRI, it is still the most easily accessible neuroimaging procedure in many centers.

### Conclusion

WMD severity of the brain is associated with osteoporosis in the elderly. This study raises the possibility of a link between ischemic CVD and osteoporosis. Osteoporosis may be a clinical manifestation of an underlying multifactorial disease process including the central nervous system. The mechanisms connecting the brain and the bones could have implications for future therapies which can better deal with osteoporosis. Better understanding of the biological linkages of the brain and bone may provide opportunities for combined therapies which may enhance bone density and limit CVD progression. Future prospective studies that establish the causal relationship between WMD and osteoporosis are necessary.
